# Process-in-Network: A Comprehensive Network Processing Approach

**DOI:** 10.3390/s120608112

**Published:** 2012-06-12

**Authors:** Gabriel Urzaiz, David Villa, Felix Villanueva, Juan Carlos Lopez

**Affiliations:** 1 Division of Engineering and Exact Sciences, Anahuac Mayab University, Carretera Merida-Progreso km.15.5, Merida, Yucatan 97310, Mexico; 2 Department of Technology and Information Systems, School of Computer Science, University of Castilla-La Mancha, Av. Universidad, 4, Ciudad Real 13071, Spain; E-Mails: david.villa@uclm.es (D.V.); felix.villanueva@uclm.es (F.V.); juancarlos.lopez@uclm.es (J.C.L.)

**Keywords:** middleware, heterogeneous, overlay, virtual QoS

## Abstract

A solid and versatile communications platform is very important in modern Ambient Intelligence (AmI) applications, which usually require the transmission of large amounts of multimedia information over a highly heterogeneous network. This article focuses on the concept of Process-in-Network (PIN), which is defined as the possibility that the network processes information as it is being transmitted, and introduces a more comprehensive approach than current network processing technologies. PIN can take advantage of waiting times in queues of routers, idle processing capacity in intermediate nodes, and the information that passes through the network.

## 1. Introduction

In a traditional computing scheme, data processing is performed in the processors, storage takes place in memory, and the network is used for communication. Over the years, there have been several different architectures and technologies, such as centralised and distributed computing, as well as primary, secondary and cache storage devices, but the vast majority of them appear to respect the general idea that the processing, storage, and communication should be performed in the processor, memory, and network, respectively.

There have not been many attempts at achieving something different, even if the attempted variant could result in significant advantages. Consider, for example, the case of Processing in Memory (PIM) [1], which modifies slightly the concept of a traditional memory and processor. The specific way of implementing PIM is by coupling the processor very closely with the memory, usually on the same chip. This approach will reduce the need for moving data, which is reflected in lower power consumption, reduced memory latency and increased bus bandwidth.

Another interesting combination to attempt is to mix the processing with the network. This concept could be called “Process-in-Network” (PIN), which is defined as the possibility that the network processes information as it is being transmitted.

The processing is performed directly in the network nodes that are found between the origin and the destination, taking advantage of waiting times in router queues, idle processing capacity in intermediate nodes, and the information that passes through the network. If a link is busy or does not meet the QoS (Quality-of-Service) requirements, and provided that there are sufficient resources in the intermediate node, it is reasonable to consider the possibility of performing processing during the time that the link is unavailable and/or fails to meet the QoS requirements.

Today's routers are very fast and they are very efficient at performing routing tasks, but this may not be enough to ensure an adequate data flow. There is still the possibility of a bottleneck. A network could become congested when the buffers are saturated due to limitations in the link capacity. Every network is likely to become congested. If a network is never congested, it may be oversized and that would imply an unjustified investment. PIN could be understood as a kind of countermeasure when the network becomes congested. There is an opportunity for PIN while the link is busy, especially in congested networks. The more congested the network is, the greater the advantages of using PIN.

These constraints (either in terms of capacity, delay, cost, power consumption, or any other factor or combination of them) are a primary motivation for seeking alternatives that optimise the transmission of information through the network. Traditional alternatives refer either to mechanisms that attempt to find the best route between the origin and destination or to find solutions that simplify the information in the input terminal nodes to reduce the burden that must be sent to the next hop.

Another important idea to consider as part of a PIN is the ability to leverage information as it flows through the network, using information fusion techniques [2], whether based on information at a node or as the result of the aggregation of several nodes. The information upon arrival to the destination is better and richer in comparison with the information that was originally sent.

The benefits of PIN concern mainly the following three factors, with the understanding that each of them can occur (or not) concurrently with the others, depending on the scenario and the specific application:

- A significant reduction in the use of the links because the information is simplified and this therefore reduces the need for network transmission. This possible simplification or reduction in the amount of information transmitted is never at the expense of a loss of semantic content;
- An enrichment of information results from a merger of processing information as it passes through the network;
- A lower requirement of time and processing at the destination end nodes results because the information reaches its destination with a pre-processing level that is achieved by its transit through the network.

Some of the features that a comprehensive network processing solution should include are the pre-processing, the data simplification, and the data enrichment functionalities. The pre-processing functionality is that information is being processed along the way and arrives at its destination with some degree of processing, instead of starting the process after it reaches the destination. Let's consider as an example an image processing application to reduce the size of an image [3]. A very simple method could be used to reduce the size of the image, consisting in dividing it in several pieces and send the pieces individually. The size reduction of the individual pieces may take place in the network.

The functionality of simplification of information is that less information is transmitted each time as a result of a simplification process. The key point is not the processing of information to provide a result but instead is involving less information to send packets as they travel over the network. Consider for example the case of an image that is sent to a destination but in a simplified manner. This scenario does not send the entire image but instead sends only a few selected points of the image. Another example could be a character recognition application for the use of a transit department [3]. The idea is to recognize characters of car plates, starting from the image that is get by means of a video surveillance camera. The camera takes real time video and delivers it to the network (either as video or as an already selected image), which performs the whole information processing before delivering the final result to the destination node (in data format) that could search the corresponding proprietary name and address in the data base. Image is being simplified as it travels through the network, and the information that the application needs is extracted in the last phase.

The information enrichment functionality consists of applying data fusion techniques for increasingly enrich information in terms of semantic content as it travels over the network. For instance, consider an Ambient Intelligence (AmI) medical application [4] and suppose that a patient wants to contact a doctor. He sends his request to the network, which provides an answer based on the information that was provided by one or more doctor-type nodes that were considered semantically related to that specific requirement.

### Some Examples to Illustrate PIN Functionality

Three examples of specific problems are presented to illustrate the proposed PIN functionality. These scenarios are very different, and were deliberately selected to show the wide variety of possible applications for PIN.

The first case is a deliberately simple scenario, with the intent of showing easily the possible application of network processing in the context of smart grids. In a power supply system for a city, data from individual consumption readings of each of the electricity meters are relevant for purposes such as billing (or perhaps attempting to learn what types of devices are connected to the network, to prevent inductive loads affecting the quality of the flow). If only the need to provide energy to a city is considered, the relevant figure is simply the total amount of energy that must be supplied. The traditional solution to this problem is to concentrate all readings in a central processor. The calculation is indeed very simple (most likely a simple sum), but the difficulty is in obtaining all of this information at the central server and possibly also in the size of the server, provided that it is capable of processing such a large volume of information. We might consider a second alternative, where instead of using a single central server, multiple servers are placed on a smaller scale with a distributed approach, which could offer some advantages over the centralised solution. However, in the case of a smart grid, we can consider an alternative in which the network itself makes the aggregation process, eliminating the need for servers.

The second example refers to the issue of collaboration in an underwater environment. Most applications for underwater environments (oceanographic data collection, environmental monitoring, navigation, tactical surveillance) use underwater vehicles that are based on inter-vehicle communications capabilities for the exchange of information and coordination purposes [5]. The enormous diversity and structural complexity of various underwater environments, such as coral reefs, coupled with adverse underwater conditions, impose a difficulty that is inherent in what the scientist can accomplish when attempting to understand and characterise the structure and functionality of the components of the reef. In an environment with a strong need for collaboration between nodes, but with important limitations in communication links, it is necessary to optimise the transmission to the fullest. PIN could be an interesting alternative, first to reduce as far as possible the need for communication, to perform compression, simplification and optimisation before a message is sent. PIN can also be a factor that helps to build relevant and complete information as it travels across a network.

We might consider yet another example. It is clear that transfers involved in a critical application of telesurgery, because human life is involved, must comply with stringent QoS requirements. QoS requirements for the primary surgeon will undoubtedly be the most strict, but a telesurgery application might also be considered for other audiences, in which they all receive the same images or video, but each could have different QoS requirements. For example, QoS requirements to other physicians who supervise and advise the surgery could be slightly less stringent than those required for the primary surgeon. For medical students who are watching the operation, the QoS requirements could be even lower. In some cases, it can be considered to be sufficient (and even desirable for reasons of clarity) to have a simplification of the original image or video, which is obtained as a result of a visioning process. Consider, for example, the case of the recognition of objects such as a tumour or an artery, along with their respective elements of location (e.g., position, orientation). If the links are limited, but as long as there are sufficient resources to process the intermediate nodes, it is reasonable to consider the possibility that the network performs certain tasks as the packet is transmitted. The network could be responsible for processing the original image or a video to suit different QoS requirements and could deliver the data to each user depending on their specific QoS requirements and network conditions at any given time.

The above descriptions definitely raise three very different scenarios but have an important element in common, which is the possibility of providing value through an implementation of PIN. It is possible that the functionality that is implemented in each case could be very different (an aggregation of abundant and widely dispersed information, a simplification of the information as it travels over the network, filtering, and enrichment of information, image processing or video to adapt to different QoS requirements); however, in all cases, an important contribution of value is identified. The benefits that are achieved may be different in each case (e.g., the elimination of servers, a reduction in the network load, enriched information and more appropriately, facilitation of collaboration in environments with severe restrictions on links); however, in all of these situations, it appears reasonable that the network can perform processing.

## 2. Related Work

There are important technologies that perform a type of network processing, but they all perform processing for very specific purposes and with a somewhat limited scope. This section briefly presents the most relevant cases.

### 2.1. Active Networks

In a conventional data communication network, routing components are passive, and routing decisions are made based only on packet header information. In contrast, active networks allow for the possibility of changes in real-time network operations, also allowing the possibility of performing network computing by using routing instructions and a user-defined process and by installing on-demand-based network services software. Active networks allow applications to adapt the infrastructure network. This paradigm improves the end-to-end performance of some types of network applications, through the delegation of the front-end implementation of tasks to network nodes [6]. Active networks also allow the ability to add computer power in the network. This scenario usually occurs within the same processor nodes but could also use general purpose processors that are externally connected as virtual routers to play the role of active co-processors, as an alternative to adding capacity to existing active network routers. A router-assistant to active nodes is presented by Larrabeiti *et al.* [7], which has the characteristics of transparency, IPv4 and IPv6 support, and complete control over layer 3 and above. An entirely middleware-based architecture is presented by Cook *et al.* [8], which addresses authentication, memory management, and interconnectivity problems that would otherwise be inherent and enables a highly functional multi-language interface for the deployment of dynamic protocols. Their results show the feasibility of an active network infrastructure implemented in middleware.

### 2.2. Overlay Networks

Amutharaj *et al.* [9] define an overlay network as a network that runs on top of another network. The overlay networks build a logical end-to-end delivery infrastructure, which is mounted on the existing transport networks. A possible drawback is that an overlay network could involve an additional cost, which refers to two main problems: duplication of features and the likelihood of adding too much overhead by encapsulation.

Overlay networks have been widely popularised in recent years because of the advantages in many areas of networks, ranging from multicasting and packet routing to the location of resources or objects in a distributed environment [10]. Overlay networks have been used to support a number of different applications: from their origins supporting file sharing, they have expanded to include more real-time applications and interactive features, such as streaming multimedia, voice over IP, and real-time gaming. Each of these applications requires different levels of QoS. For example, a file transfer application requires a path that has more bandwidth available, while real-time interactive applications have latency requirements (delay) and jitter [11]. The overlay networks can help to solve some problems, in particular, end to end QoS [12].

The Peer-to-Peer (P2P) architecture is often used to implement overlay networks. Unlike what occurs in a client-server architecture, nodes in a P2P network behave as equals and can act simultaneously as clients or as servers for the other nodes in the network. An extensive compilation and comparison of P2P overlay network models is provided by Lua *et al.* [13]. Depending on the optimisation technique used, the approaches that seek to provide QoS by P2P overlay networks can be classified into two main groups:

- Approaches targeting path optimisation. There are several proposals that seek to make optimal use of the network, by finding the best overlay network topology to minimise the overhead. Within this group are the following solutions: additional auxiliary nodes [14], hierarchy of nodes [15], organisational groups [16], simplification of the network [17], replication [18], semantics/users [19], symbiotic networks [20], multiple paths [21], a variety of routes [22], adaptive/unstructured approach [23], bandwidth reservation [12], flexibility in the underlying layer [8], and traffic prioritisation [24];
- Approaches focusing on information optimisation. This approach attempts to perform some processing of information to make a simplification. Within this group are the following solutions: distributed infrastructure [25], simplification of goods [26], adaptation based on context [27] or QoS requirements [28], and urgency-based scheduling [29]. Although the size of the problem has been limited due to the fact that the amount of information has been reduced, this type of solution is still exposed to the problems that occur in the network.

### 2.3. In-Network Processing (i-NP)

There is also a history of network processing for *ad-hoc* networks, specifically for sensor networks, for which energy consumption is one of the most critical problems. One of the alternatives is called in-Network Processing (i-NP), which is to seek energy savings by reducing the number of transmissions.

The Directed-Diffusion [30] and the LEACH [31] protocols have been proposed to extend the lifetime of energy-constrained wireless sensor networks. Several enhancements to these protocols [32–34] and also some other approaches were designed with the same idea in mind, such as PER [35], a power-saving hierarchical routing protocol.

An i-NP approach provides greater efficiency in energy consumption than the traditional centralised server model, in which nodes simply collect and send data to a central powerful node. This i-NP approach seldom considers network issues, for example, the instantaneous load on the network or aspects of the service quality. The intention is to minimise the amount of information before sending it, to minimise the power consumption. Many of the common applications of sensor networks require data processing. The complexity of the process varies significantly from one application to another, even within the same application.

Download processing has been studied in the context of low-power portable systems. A lower bound on the computation time is derived by Ayaso *et al.* [36]. This bound must be satisfied by any algorithm used by the nodes to communicate and compute, so that the mean square error in the nodes' estimate is within a given interval around zero.

In wireless sensor networks, one is not interested in downloading all of the data from all of the sensors; instead, there is an interest in simply collecting from a sink node a relevant function of the sensor measurements. An interesting study of the maximum rate at which functions of sensor measurements can be computed and communicated to the sink node is presented by Giridhar *et al.* [37].

i-NP has been explored by several authors. An approach of aggregation for wireless sensor networks with multiple deposits is proposed by Son *et al.* [38]. The experimental results show a reduction in the number of transmissions and, thus, a savings in energy. In-network processing is also explored by Kamath *et al.* [39] and describes a protocol for pipelined computation in a structure-free random multihop wireless network. A network-level architecture for distributed sensor systems is presented by Tsiatsis *et al.* [40], which performs i-NP, whereas heterogeneous nodes are organised in a hierarchical structure dictated by their computational capabilities. The presence of high-performance nodes in the middle of a sea of resource-constrained nodes exposes new commitments for the efficient implementation of applications across the network. Experiments show that, even for a relatively low node density with limited resources compared to high-performance nodes, there are some performance gains for a hierarchical heterogeneous system compared with a homogeneous system. There are some tradeoffs between the run-time implementation, the accuracy of the output produced, and the total energy consumption in the network.

## 3. Purpose

The detailed analysis of the state of the art leaves one feeling that the issue of network processing has been addressed so far in only a very timid and tangential manner, which suggests that there is a need for a more direct and comprehensive coverage of the subject (Table 1).

Active networks perform processing with the primary aim of modifying the operation of the network at any given time. While also allowing the inclusion of user code in the network nodes, this scenario is accomplished only as a possibility that the user opens and is not offered in any way as an additional functionality provided by the network. Overlay networks could be used to optimise the flow of information in the underlying network. Most of the existing approaches belong to the first group (route optimisation), which refers mostly to routing issues, while the existing approaches that are dedicated to the second method (information optimisation) are limited to a simple process of simplification of the information in the terminal nodes. The i-NP technology is a step closer to the concept of processing in the network that is presented here, but its purpose and approach are again limited. It is focused mainly on minimising energy consumption, for which it relies mainly on a reduction in the amount of information that must be sent. Its current applicability is limited mainly to the field of wireless sensor networks. This approach rarely considers the possibility of enriching the information as it passes through the network, and it is not common to account for issues concerning the network, such as instantaneous load conditions on the network or QoS. A detailed review of the history shows that existing approaches are isolated and limited, which opens the possibility of developing the subject in a comprehensive and direct way (Table 2).

The terms “limited” and “rarely” mean that existing solutions of this alternative offer very little support for this specific functionality. Some features could be implemented with existing approaches, but PIN has a larger vision and may be more suitable for complex applications.

We are convinced that network processing can provide significant value in certain environments. The development using existing techniques is possible, but it would involve a major change in their original design, especially for complex applications. We think that a new model with a broader approach could be used in a more natural way and may be more suitable. PIN was conceived as a more comprehensive solution than the existing solutions so far.

The main objective of PIN is much wider than the purpose of existing approaches, to include important features such as heterogeneity, QoS profiles, and the benefits of an object-oriented approach.

IDM/PIN could deal with very heterogeneous networks and devices, including data link and network technologies. It is also a transport-independent solution. It also allows the definition of QoS profiles that affect the behavior of the virtual network, based on details of the application or user preferences. For example, to prioritize which flow is processed in the routers depending on the identity of the user that sends or receives the data.

IDM/PIN routers are objects, and the processing components in the routers are also objects. This is very important because of the following reasons:

- It can seamlessly integrate logic implemented in arbitrary devices (including FPGAs), and therefore it is possible to implement a hardware IDM router with PIN capabilities;
- It allows deployment of upgrades, migration of routers, versions, fault tolerance, and all services that are available when using an object-oriented middleware (such as ZeroC ICE);
- It is possible to control network processing (execution of partial phases in the routers) using different levels of granularity. It is possible to control which flows, clients, connections, or even object methods PIN is applied to. This could be done by configuring the components that are deployed in the routers, both dynamically and remotely because they are also remote objects.

The overall objective of this work is to design and develop a comprehensive PIN mechanism with a wide range of applications and a broad functionality. The processing functions of the network proposed here require the development of specific techniques in various areas of network engineering, such as routing algorithms, deployment, QoS, and the application of statistical or soft computing techniques. Based on these possibilities, the following specific objectives arise:

- Provide support for a wide variety of equipment, protocols and technologies (heterogeneous networks) for both structured networks (traditional networks) and for environments with no structure (*ad-hoc* networks), limiting the ability to manage the links (in terms of the capacity, delay, cost, or any other factor or combination of these);
- Provide the ability to handle large volumes of information and a wide range of services (text, audio, image, video), considering at all times the inherent complexity in the treatment of each of them;
- Consider all the time QoS aspects and provide the means necessary for the participation of users with different QoS requirements and / or quality of experience (QoE);
- Implement elements of networking (with the end-user application, with routing algorithms, or with any other element that is necessary), to conduct efficiently and effectively the processing functions of the network.

## 4. Methodology

This proposal is based on the concepts of Inter-Domain Messaging (IDM) [41] and Virtual Quality-of-service Networks (VQN) [42]. IDM is a novel solution for transporting messages in a heterogeneous environment. IDM was designed as a general purpose protocol for providing a data transport service end-to-end, which is independent of any network technology or protocol. IDM uses its own addressing and routing mechanisms. The communication model is based on object invocations, which provide many valuable advantages, such as a full location transparency and the possibility of deploying specific application code in a very simple manner, even better performance when compared to traditional IP-based solutions. Although IDM adds overhead, it could be a more efficient solution in many cases because it is a cross-layer protocol.

VQN is an overlay network that uses IDM as a basis. It is implemented as a distributed application using object-oriented middleware for distributed systems. The original idea for the VQN model is to develop a mechanism that provides performance with Quality of Service (QoS) to a network that naturally does not have this structure, but its usefulness is not limited only to operations that are related to the network itself; it can also be used to provide additional functionality related to the application, such as information processing, semantic collaboration, and others.

VQN and PIN have intrinsic capabilities for dynamic adaptation to network conditions. A feedback mechanism could be implemented in order to dynamically adjust Quality-of-Service (QoS) or Quality-of-Experience (QoE) for an specific user at the destination node. A feedback mechanism may be included as part of the PIN mechanism and make all the necessary QoS/QoE adjustments based on the feedback line. VQN has already several mechanisms (*i.e.*, fuzzy logic and forecasts) that may be helpful when implementing this feedback mechanism.

**Figure 1.**
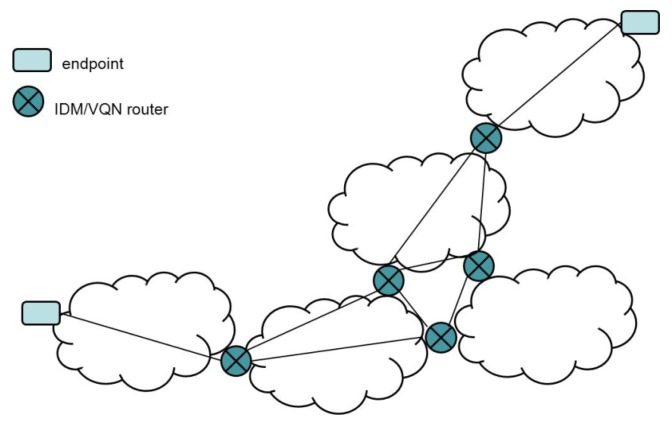
An IDM/VQN inter-network.

Notably, the IDM/VQN routers are virtual. Commercial (physical) routers are usually far from the reach of the distributed applications user. In most cases, they are dedicated to running specific protocols to provide an efficient forwarding process, and it is not possible to use them to deploy specific applications code. IDM/VQN makes deployment feasible and, thus, opens the possibility of implementing a PIN in a real environment (Figure 1).

**Figure 2.**
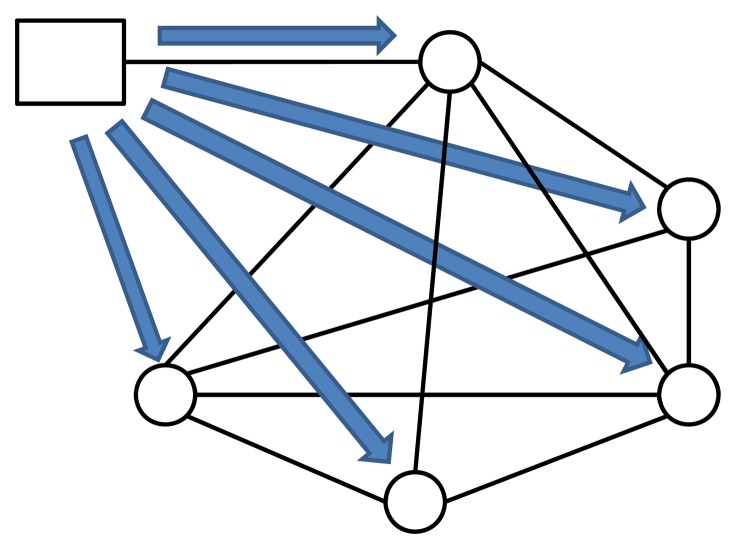
Code deployment process.

The code deployment process is dynamic and for a real implementation it is based on ZeroC IcePatch2 deployment capabilities for secure replication of a directory tree. The server manages the file system directory containing the software to be distributed to the clients. The server transmits (Figure 2) the files to the client, which recreates the data directory and its contents on the client side, replacing the old files with the new ones. IcePatch2 transfer rates are comparable to File Transfer Protocol (FTP).

**Figure 3.**
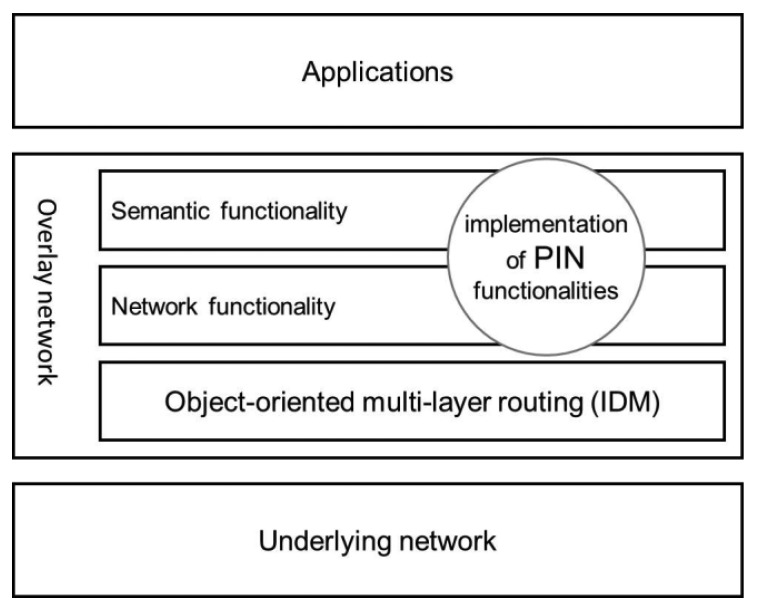
Implementation of PIN functionalities within the VQN model.

Our proposal is to use the IDM/VQN platform to implement PIN, taking advantage of all of the features mentioned above (transparency, deployment, and performance) and others. The processing logic that depends on the application is deployed to the routers as an implementation of distributed objects. PIN is integrated into the IDM/VQN infrastructure in a very natural way because the routers themselves and all of the other services are also distributed objects. Every IDM/VQN router is capable of implementing PIN functionalities, either as a part of the semantics or the network sublayer or even as a combination of both (Figure 3).

Initially, three PIN functionalities are considered for the model: pre-processing, simplification of information, and information enrichment. These features can be upgraded and supplemented as the model evolves.

### 4.1. PIN Modes of Operation

Two modes of operation are considered for the model: the PIN process on a packet-by-packet (PbP) basis and the PIN process on a block-by-block (BbB) basis. A packet is a single unit of information, and a block is defined as a group of one or more packets that could be processed as a whole.

Both modes of operation are implemented by using IDM as the transport. The PIN is implemented by placing distributed objects into intermediate nodes. This scenario simplifies the deployment and updates the PIN phases as objects. A PIN intermediate node receives a message that transports an invocation (in one or more packets). As soon as it is complete, it can execute it at its local PIN object, generating a new invocation message that can be sent to the next node or again to the local PIN object to perform another phase.

Importantly, the packetisation and blocking processes are performed as a part of the PIN function within the semantic sublayer of the overlay network, which allows the application to remain unchanged. The phases are described in the distributed application definition, and they depend on the specific application semantics. The PIN can be considered to be an application-oriented protocol. It uses invocation messages instead of data packet messages used by traditional network protocols.

### 4.2. PIN Requirements

PIN imposes two requirements on the information that is sent through the network:

- Information is divided into packets that could either be processed independently from the others (*i.e.*, processed on a PbP basis) or be processed as a group (*i.e.*, processed on a BbB basis) that can be considered independent from the others;
- Information processing can be divided into phases.

In addition, there are two more requirements for the nodes in the network:

- Network nodes can perform some processing;
- The nodes must have sufficient storage capacity to process at least one packet (in the case of a PbP basis) or at least a full block (in the case of a BbB basis).

### 4.3. The PIN Process in the Case of Packet-by-Packet (PbP) Basis

The PIN process on a packet-by-packet basis includes four steps. The first step determines, for the application, the information unit and the corresponding size. An information unit can be processed independently from the other packets, and the different processing phases (identified with numbers from 0 to n, where 0 means “no processing has been performed” and n means that processing has finished).

The second step is to add an additional “process phase” field to each packet that is sent to the network. It is expected that all of the packets are sent with a zero in this field, which is not a restriction in any way. It could be possible that a source node performs some processing before sending the packet.

The third step is that every intermediate node processes the packets (and updates the “process phase” field accordingly) instead of sending it to the transmission queue. The PIN function is continuously monitoring the queue length to determine the best moment to stop processing and putting the packet in the transmission queue.

The fourth step is performed at the destination end node and finishes the processing that was not performed in the network.

**Figure 4.**
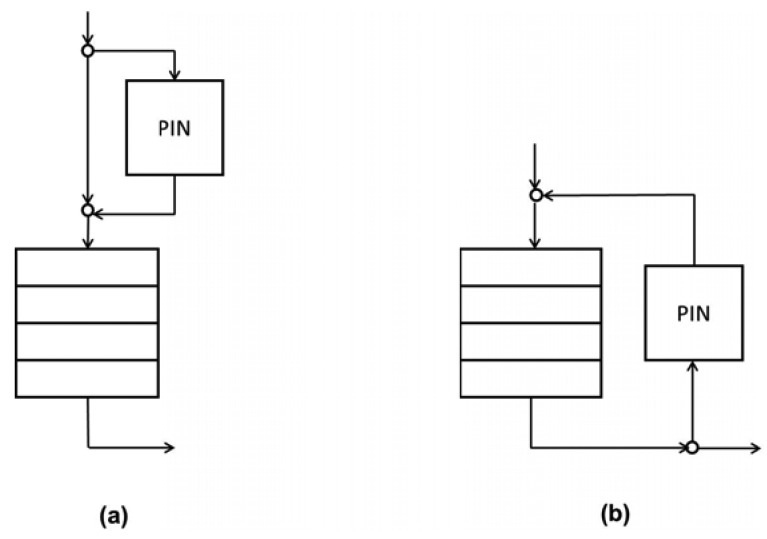
Moments in the queue for initiating the PIN on a packet-by-packet basis.

There are two moments in the queue at which the PIN on a PbP basis could be triggered. One option (Figure 4(a)) initiates the PIN at the queue input depending on the queue length and other possible conditions (e.g., the processing phase). In this case, the PIN is performed only for the arriving packet. Another possibility (Figure 4(b)) is to perform the PIN for all of the packets in the queue. The PIN could be performed using either one or the other option or even combining both options.

### 4.4. The PIN Process in the Case of a Block-by-Block (BbB) Basis)

**Figure 5.**
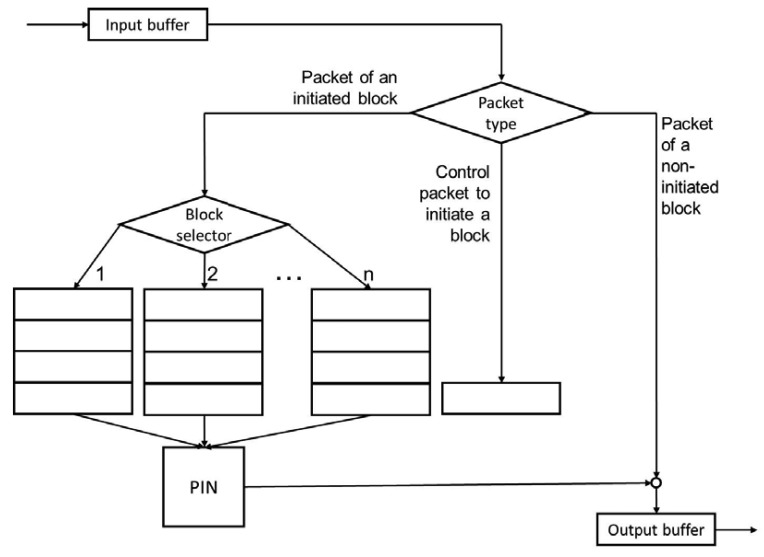
The process on a block-by-block basis.

Four steps are considered for a PIN process on a block-by-block basis (Figure 5). The first step is to identify the building blocks of the information. For example, in the case of a video, we can consider that each image is independent of the others and can be treated separately without affecting the others.

The second step is to add a control packet at the beginning of the transmission of a block, which indicates the block identifier and the total number of packets that are part of the block. This scenario is similar to a session establishment mechanism. Each time that a node receives a packet PIN control, it opens the corresponding block and becomes ready to receive packets for that block.

The third step is to add a field of “block ID” to each packet to identify packages that are part of the same block.

The fourth step is to initiate the process of the block as soon as it is complete. A node can have opened several blocks but only begins PIN processing when all of the packets are received for that block.

## 5. Evaluation

The evaluation process included two types of tests. First, we conducted some experiments on a network simulator to obtain numbers about the opportunities for PINs and to demonstrate PIN feasibility. Second, we built the first version of a software prototype that validates PIN functionality and can be used as a helping tool for the future development of the model.

PIN has been conceived as a more comprehensive network processing approach than the existing approaches so far, including some features that may not have been implemented in previous solutions, and therefore it could be difficult to establish a fair comparison scenario on a quantitative basis, due to the fact that functionalities may not be implemented in every approach. The evaluation process was designed to demonstrate that the idea of network processing could provide important benefits, with the understanding that similar results could be obtained if the same functionality was implemented by any other network processing solution.

### 5.1. Simulation Tests

All of the simulation tests were performed on an IBM-compatible PC with an Intel Atom@1.6 GHz processor, running Debian GNU/Linux OS with a g++ compiler and OMNeT++ v4.1 [43].

**Figure 6.**
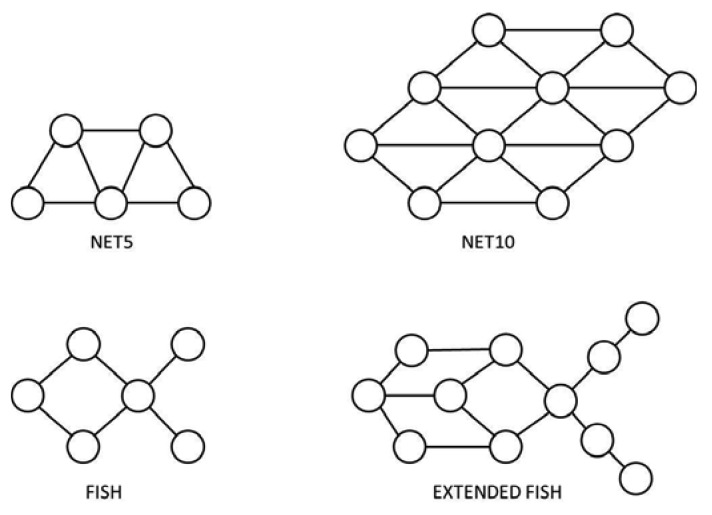
Network topologies used in the experiments.

We used four different network topologies (Net5, Net10, Fish, and Extended Fish), which are represented in Figure 6. These network topologies were selected in order to be able to analyze the effect of congestion due either to network size or to the presence of bottlenecks. The Net5 and Fish topologies correspond to small networks (five and six nodes respectively) while Net10 and Extended Fish topologies define relatively bigger networks (10 and 11 nodes respectively). Every node in Net5 and Net10 is well communicated (almost full-mesh topologies), while Fish and Extended Fish topologies involve significant bottlenecks.

**Figure 7.**
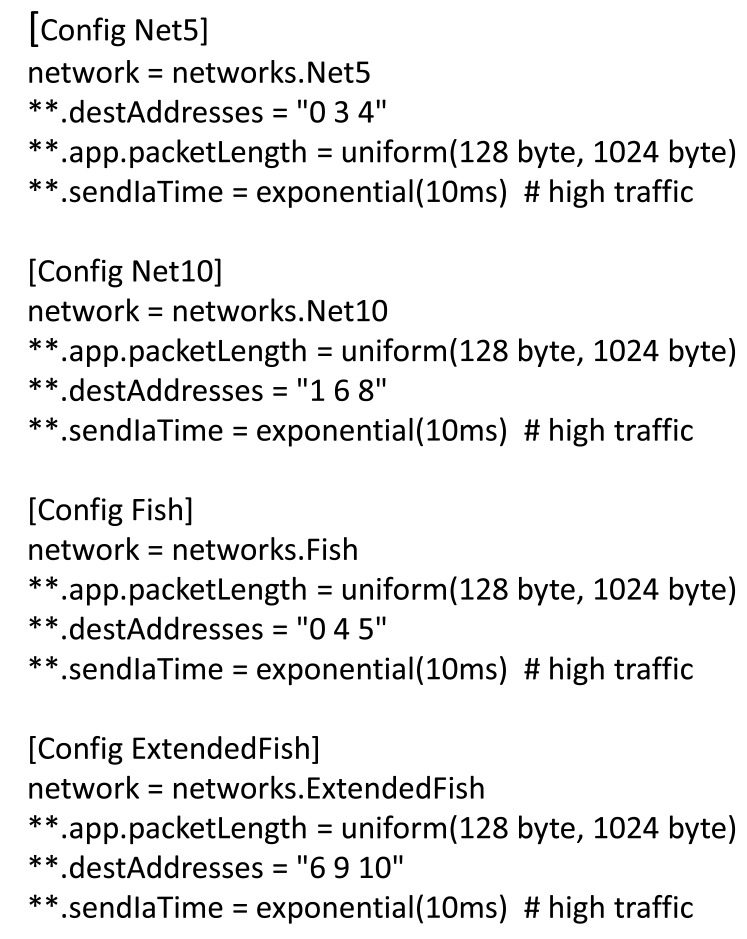
Characteristics of the traffic that was injected.

The experiment considered relatively small networks, but it was enough to provide important information about the effect of network congestion due to network size and the presence of bottlenecks. We expect that the effect would be magnified if more nodes were used in further experiments, that could also be used to provide information about scalability. A high volume of traffic (Figure 7) was generated automatically. In order to promote network congestion, we concentrated all the generated traffic in just three destination nodes.

All simulation tests were limited to 100 s.

**Figure 8.**
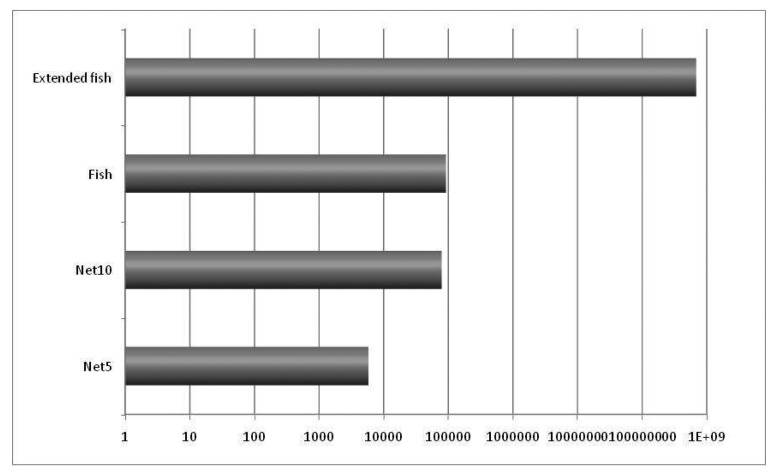
Opportunity for PIN (in seconds, logarithmic scale).

The main idea in the first experiment was to obtain preliminary numbers about the lengths of the queues in the nodes and the average time in each queue, to determine the opportunity for PIN, which is calculated by multiplying the number of queued packets (qlen count) times the average queueing time (qlen time avg). The results are graphically presented in Figure 8.

It should be noticed that opportunity for PIN increases with the network size (comparing Net10 to Net5, and Extended Fish to Fish) and with the presence of bottlenecks (comparing Fish and Extended Fish to Net5 and Net10).

This opportunity for the PIN number indicates how many packets and for how long they stay in the queues. This scenario means that it is possible to perform processing during that time. This first experiment is useful for determining how many packets there are and how much time they stay in the queues and, therefore, the packets that are susceptible to being processed in the network. The actual network processing opportunity will depend not only on the fact that a certain amount of packets are in the queues but also on the real process capacity that is available at each intermediate node, which could be used to process the queued packets while they are waiting to be transmitted.

For the second experiment, we wrote some simulation code to demonstrate the PIN feasibility, specifically for the functionalities of pre-processing and information simplification on a packet-to-packet basis.

To validate these functionalities, three corresponding indexes were defined.

- The Pre-Processing index (PP index) was expressed as a percentage. A reference value of 0% means that no pre-processing was performed by the network. A value of 100% means that information arrives completely processed;
- The Simplification-of-Information index (SI index) is expressed as a percentage and is calculated as a function of the total size (expressed in kbps) of the original information that is to be transmitted from the source node. The reference value is 0%, which means that the complete information is transmitted in the network. A value close to 100% means that information is simplified maximally, and therefore, almost none of it is transmitted over the network;
- The Information-Enrichment index (IE index) is expressed as a percentage. It is calculated as a function of the total original content of the information in the original message. For the original network, a 100% value is considered to mean that the original message contains itself the whole information content that must be transmitted. A value of less than 100% would mean that some content has been lost, and a value that is higher than 100% would mean that information has been enriched.

**Figure 9.**
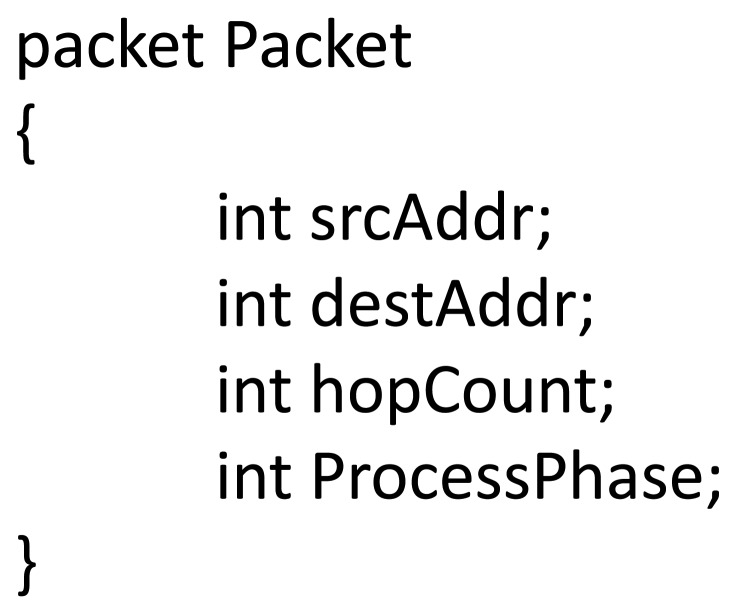
Definition of the ProcessPhase field.

A simple data processing application was simulated with eleven processing phases (0 to 10) that were defined, and every packet at the origin was sent with a zero value. The intermediate nodes performed PIN whenever the queue length was greater or equal to three packets, and the duration of each processing phase was 3 seconds. First, a new ProcessPhase field is added to the packet definition Figure 9).

A new PINalarm field was also added to measure the preprocessing percentage. Values were captured whenever a packet was transmitted. In the application handlemessage class, the initial value for ProcessPhase is set to zero for every generated packet. A new checkpoint is added before a packet is added to the queue.

**Figure 10.**
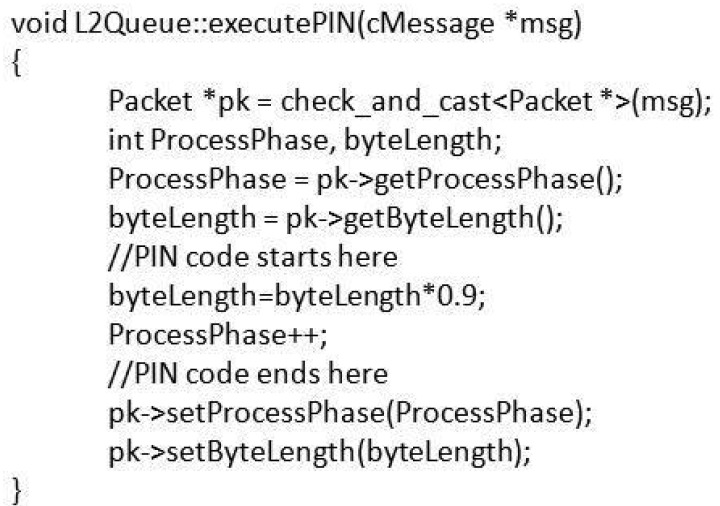
The ExecutePIN module.

PIN is initiated if the queue length is higher than 3 and the ProcessPhase is less than 10). We wrote a dummy ExecutePIN module (Figure 10), which considers that each phase reduces the byte length by 10 percent.

We first attempted to send the packet to a different module (a PIN application module was added to the Network Description (ned) configuration, which is the topology description language for OMNET++), but we finally decided not to use this option because of the additional time that was added. We decided to include the PIN code within the queue module code.

The results (Table 3) show that PIN reduced the queues (a lower number of queued packets and a lower average queueing time in most cases), and because of the reduced queuing time, there is an opportunity for having PIN at a zero cost.

The numbers in the last column (Maximum PIN time for cost = 0) were calculated by comparing how many packets and for how long they stay in the queues (qlen count multiplied by qlen time), between runs without and with PIN activated.

The qlen count increment observed in the last case (extended fish) is because of the congested nodes. PIN helped to reactivate the network flow, and therefore, more packets started to move between the nodes, which caused this increment. The best PIN numbers (the preprocessing percentage and the maximum PIN time for a zero cost) were achieved in the most congested network. PIN adapts to the network conditions at every moment. PIN does almost nothing in a non-congested network, but as the load increases, it appears to compensate for the waiting times at the routers.

**Figure 11.**
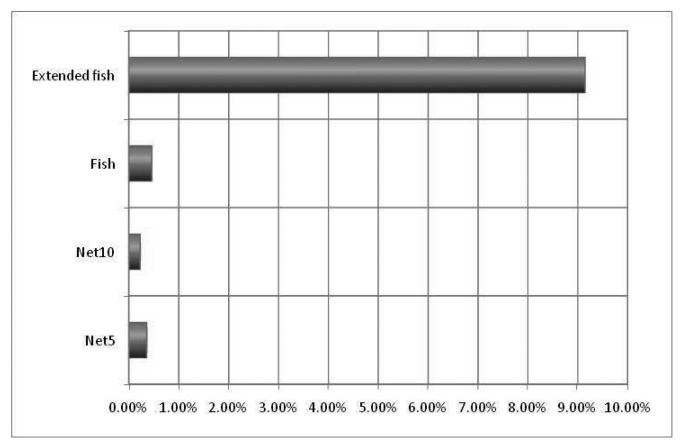
The pre-processing index (percentage).

The experiment was also useful to show that information was delivered with a certain degree of pre-processing. The Pre-Processing Index numbers are shown in Figure 11.

Our experiments show that 10% pre-processing levels could be obtained, but this number is with regards to the specific topology and network conditions in the experiment. In a real scenario this number may be less, but also conceivably much higher (even 100%) depending on the topology, traffic, and network conditions.

Only the pre-processing and simplification of information functionalities were simulated in the experiment. Information fusion techniques were not included in the experiment. It is expected that this element could contribute significantly to enhance the PIN benefit numbers.

### 5.2. Software Prototype

The software prototype was written in the C++ programming language complemented by a distributed application by means of object-oriented middleware for distributed applications (*i.e.*, ZeroC ICE [40]). Development was accomplished on an IBM-compatible computer with an Intel P4@2.24 GHz processor, with a Windows XP Professional operating system with SP3, Visual Studio 2005 SP1 and ZeroC ICE v3.3.0. Prototype tests included nodes with Windows platforms similar to the development environment as well as Debian GNU/Linux 6.0 (squeeze) nodes with ZeroC ICE v3.3.1-12.

The implementation of the VQN model included the following features: QoS-aware, multihop, forecasts, and Fuzzy Logic. The prototype included the three initial PIN functionalities (pre-processing, simplification of information, and information enrichment).

**Figure 12.**
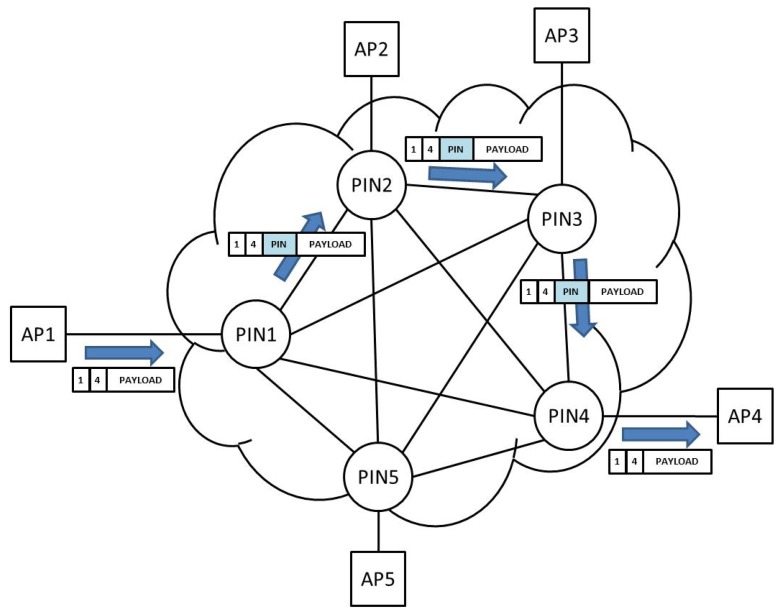
Software prototype test scenario.

A simple file transfer application was implemented. A 5-node full-mesh topology was considered for the test scenario (Figure 12). Connectivity between nodes was based on ZeroC ICE interfaces, allowing the connection of nodes by means of input buffers. Any application node (for example AP1) was able to request a file transfer from a remote application (for example AP4). As soon as the request is received in AP4 it sends back the requested file contents to AP1.

## 6. Conclusions and Future Work

Process-in-network (PIN) adds value when solving important problems. The review of the background shows an opportunity to contribute with a more comprehensive approach than the solutions that have been offered so far.

Simulation results show an opportunity for PIN and demonstrate the feasibility of PIN. The software prototype is currently being used to implement proofs of concept for specific application environments. Initial scenarios include heterogeneous intelligent networks and Ambient Intelligence (AmI) applications.

PIN can be used in AmI to provide a solid, efficient and functional communication platform to support highly demanding applications that require the transmission of large amounts of multimedia information in a heterogeneous network.

## Figures and Tables

**Table 1.** Main network processing approaches to date and PIN.

**Approach**	**Main objective**
Active networks	Modify the operation of the network at any given time
Overlay networks	May be used (a) to optimise the flow of information in the underlying network, or (b) to perform a simple process of simplification of the information in the terminal nodes
In-Network Processing	Minimising the energy consumption by reducing the amount of information that must be sent
PIN	Take advantage of the waiting times in the queues of routers, the idle processing capacity in the intermediate nodes, and the information that passes through the network

**Table 2.** Comparison of PIN with previous approaches.

	**Active networks**	**Overlay networks**	**i-NP**	**PIN**
Real-time changes	Yes	Rarely	No	Yes
Performance enhancement	Limited	Yes	Limited	Yes
Functionality enhancement	No	Yes	No	Yes
Information simplification	No	Rarely	Yes	Yes
Information enrichment	No	Rarely	Rarely	Yes
Less load at the destination nodes	No	Rarely	No	Yes
Network-aware	Yes	Rarely	Rarely	Yes
QoS-aware	No	Rarely	Rarely	Yes
Application-aware	No	Rarely	No	Yes
Type of nodes	Physical	Virtual/Physical	Physical	Virtual/Physical
Range of application	Limited	Wide	Limited	Wide
Volume of information	High	High	Limited	High
Range of services	Wide	Wide	Limited	Wide

**Table 3.** PIN benefits and costs.

**Network name**	**Qlen count difference: [packets (percentage)]**	**Qlen time avg difference: [seconds (percentage)]**	**Maximum PIN time for cost = 0 [seconds per packet]**
Net5	−2 (−1%)	−0.09317 (−47%)	0.09319
Net10	−460 (−36%)	−0.006196 (−10%)	0.06423
Fish	−674 (−61%)	−0.16102 (−19%)	0.16613
Extended fish	+31,870 (+726%)	−787.61389 (−50%)	682.00842
